# Establishment of Clinical Prediction Model Based on the Study of Risk Factors of Stroke in Patients With Type 2 Diabetes Mellitus

**DOI:** 10.3389/fendo.2020.00559

**Published:** 2020-08-25

**Authors:** Rong Shi, Taotao Zhang, Hui Sun, Fan Hu

**Affiliations:** School of Public Health, Shanghai University of Traditional Chinese Medicine, Shanghai, China

**Keywords:** stroke, type 2 diabetes mellitus, risk factor, prediction model, external validation, dynamic nomogram

## Abstract

**Purpose:** Stroke has sparked global concern as it seriously threatens people's life, bringing about dramatic health burdens on patients, especially for type 2 diabetes mellitus (T2DM) patients. Therefore, a risk scoring model is urgently valuable for T2DM patients to predict the risk of stroke incidence and for positive health intervention.

**Methods:** We randomly divided 4,335 T2DM patients into two groups, training set (*n* = 3,252) and validation set (*n* = 1,083), at the ratio of 3:1. Characteristic variables were then selected based on the data of training set through least absolute shrinkage and selection operator regression. Three models were established to verify predictive ability. Foundation model was composed of basic information and physical indicators. Biochemical model consisted of biochemical indexes. Integrated model combined the above two models. Data of three models were then put into logistic regression analysis to form nomogram prediction models. Tools including *C* index, calibration plot, and curve analysis were implemented to test discrimination, calibration, and clinical use. To select the best predicting model, net reclassification improvement (NRI) and integrated discrimination improvement (IDI) were put into effect.

**Results:** Eleven risk factors were determined, including age, duration of T2DM, estimated glomerular filtration rate, systolic blood pressure, diastolic blood pressure, low-density lipoprotein, high-density lipoprotein, triglyceride, body mass index, uric acid, and glycosylated hemoglobin A_1c_, all with significant *P*-values through logistic regression analysis. In the training set, areas under the curve of three models were 0.810, 0.819, and 0.884, whereas in the validation set, they were 0.836, 0.832, and 0.909. Through calibration plot, the S:P values in the training set were 0.836, 0.754, and 0.621 and were 0.918, 0.682, and 0.666 separately in the validation set. In terms of the decision curve analysis, the risk thresholds were, respectively, 8–73%, 8–98%, and 8%~ in the training set and 8–70%, 8–90%, and 8–95% in the validation set. With the aid of NRI and IDI, integrated model is proved to be the best model in training set and validation set. Besides, internal validation was conducted on all the subjects in this study, and the *C* index was 0.890 (0.873–0.907).

**Conclusion:** This study established a model predicting risk of stroke for T2DM patients through a community-based survey.

## Introduction

Type 2 diabetes mellitus (T2DM), accounting for ~90% total diabetes cases, is one of the most threatening non-communicable chronic diseases. Data from the latest IDF Diabetes Atlas showed that the number of adults aged 20–79 years in the world suffering from diabetes was ~463 million in 2019. Diabetes mellitus (DM) is a great growing public health burden in China as the prevalence estimated at 11.6%, whereas that of prediabetes was ~50.1% (1).

Stroke, as one of the macrovascular complications related to DM, results in extracranial carotid artery disease and intracranial large and small vessel diseases and includes clinical characteristics ranging from asymptomatic carotid artery occlusion or cerebral small vessel disease to transient ischemic attack and hemorrhagic and ischemic stroke (2). Stroke has been acknowledged in the form of a major issue in public health contributing to morbidity and mortality worldwide. According to the *Atlas of Heart Disease and Stroke* released by the World Health Organization, stroke is the third cause of death (ranks after myocardial infarction and cancer) in the world, and every year ~17 million people die of cardiovascular diseases (CVDs) particularly attributed to heart attacks and strokes.

As one of the related complications of DM, stroke is the condition different from DM but with many common aspects (3). Nearly all types of stroke are known to be influenced by DM, including large artery stroke, lacunar stroke, intracerebral hemorrhage, and embolic stroke (4). Considerable prospective studies have indicated that, in comparison with non-diabetic population, patients with diabetes are at a higher risk of stroke among the western population (5–8). A Chinese hospital study based on 2,532 hospitalized patients with a first stroke showed that diabetes had a remarkable frequency of strokes than non-diabetics (9). In contrast to non-diabetics, the risk of stroke of people with DM is 2.5–3.6 times higher (4). Through a prospective observational study including 210 acute stroke patients, patients with DM were proved to shoulder the huger burden with poorer outcome brought by acute stroke compared with non-diabetic patients (3). According to statistics, 80% of DM patients eventually died of macrovascular complications (10). Accordingly, risk factors of stroke for T2DM patients urgently need to be determined.

Related studies of T2DM patients with stroke have provided evidence for us to identify corresponding risk factors. Through studies on diabetes and stroke, Wang et al. (11), Li et al. (12), and Bos et al. (13) stated that gender, estimated glomerular filtration rate (eGFR), duration of T2DM (course), postprandial blood glucose (PBG), fasting blood glucose (FBG), glycosylated hemoglobin A_1c_ (HbA_1c_), systolic blood pressure (SBP), diastolic blood pressure (DBP), age, low-density lipoprotein cholesterol (LDL-C), high-density lipoprotein cholesterol (HDL-C), total cholesterol (TC), triglyceride (TG), body mass index (BMI), and uric acid (UA) are risk factors for stroke among T2DM patients. Based on the previous studies and community survey in this study, we involved basic information indicators including gender, age, course, BMI, SBP, DBP, and family history of DM; disease record information including hypertension, hyperlipemia, and microvascular disease; lifestyle factors containing smoking and alcohol; and biochemical indicators including FBG, PBG, HbA_1c_, TC, TG, HDL-C, LDL-C, blood urea nitrogen (BUN), UA, eGFR, and the ratio of urinary microalbumin to uric creatinine (ACR) in this study.

At present, many studies on T2DM or stroke describe risk factors of stroke and T2DM patients, respectively. The study combined the two diseases and aimed to find out risk factors of stroke for T2DM patients.

This study aimed to build a simple, convenient, and efficient prediction model because of the main risk factors affecting stroke for T2DM patients. In this study, three nomogram plots were demonstrated, and the most predictive, accurate, and effective one was found through net reclassification improvement (NRI) and integrated discrimination improvement (IDI). At the same time, we also developed an online application for predicting T2DM patients with stroke based on the nomogram plot. The work can be used for clinically evaluating T2DM patients to assess the risk of stroke incidence for them.

## Materials and Methods

### Patients

We worked with Shanghai University of Traditional Chinese Medicine–affiliated community health center hospitals for this study. From September 2014 to September 2019, we conducted baseline and follow-up study on all the patients in the seven communities including Community of Huamu, Community of Jinyang, Community of Sanlin, Community of Siping, Community of Yinhang, Community of Daqiao, and Community of Jiangpu in Shanghai and finally included 4,335 subjects in this study. Subjects were determined based on their medical history information. Patients with T2DM with a history of stroke were valid to be involved in this study. Questionnaire survey, physical examination, and biochemical examination contained values of each influencing factor in this study, which were crucial for forming results. In order not to affect the model establishment and results, accordingly, for data screening, we checked the missing values at the beginning. Patients with any lack of needed information would be excluded. After obtaining all the data and comparing the various data values in the population, subjects would be eliminated with any abnormal value of influencing factors. With exclusion of invalid questionnaires and those without complete information from all the collected questionnaires, we eventually involved 4,335 subjects in the study. Before enrolling the subjects in this study, we received written informed consent from all of them.

### Procedure

We performed survey, including questionnaire surveys, physical examination, and biochemical test, and investigated all T2DM patients in seven communities with support from affiliated community health centers and central hospitals. All the researchers and investigators involved in the survey were well-trained and qualified to ensure standardization and scientific rigor in the procedure. A structured questionnaire survey was composed of social demographic characteristics, lifestyle factors, DM status, disease history, and drug history (lipid-lowering, blood pressure-lowering, aspirin, and insulin). Besides, to determine the subjects precisely, we checked the electronic medical records of all the participants for filtering. Patients with T2DM were determined as the initial population. The diagnosis of T2DM was in accordance with the criteria defined by the World Health Organization in 1999 (14). Patients with stroke were then determined through rigorous screening of medical records to ensure validity for this study and were finally included.

All the physical indicators were measured with standard electronic devices. Systolic blood pressure and DBP were measured in standard sitting with OMRON blood pressure monitors. According to the Guidelines for the Prevention and Treatment of T2DM in China, BMI was calculated with weight (in kilograms) divided by square height (in meters squared). Biochemical indexes included FBG, PBG, HbA_1c_, TC, TG, HDL-C, LDL-C, BUN, UA, and ACR. Estimated glomerular filtration rate was computed according to serum creatinine, age, and gender according to Modification of Diet in Renal Disease Trial. To test blood indicators, all the participants need to keep fasting for at least 10 h and took the examination at 7 in the morning. Two hours after the meal, urine was collected from participants for glycosuria measurement. All the blood samples were required to be taken for the operation of *in situ* centrifugation within 30 min after collection and stored in refrigerators at −80°C for further study. All the samples were at once sent to hematology department of Ruijin Hospital Affiliated to Shanghai Jiaotong University and community health centers and central hospitals affiliated to Shanghai University of Traditional Chinese Medicine for testing after the scientific operation. Urine-related biochemical indicators were analyzed by uritest-500b (URIT, China).

### Statistical Analysis

Through the community survey, we collected 4,335 T2DM patients, including 2,504 female patients and 1,831 male patients. With the aid of R software (version 3.6.2; https://www.R-project.org), we randomly divided patients into two groups, training set (*n* = 3,252) and validation set (*n* = 1,083) for external validation at a theoretical ratio of 3:1 (15). In the first step, we used data of the training set and took the least absolute shrinkage and selection operator (LASSO) regression method to analyze the data. Least absolute shrinkage and selection operator are a method applied for data dimensional reduction. Besides, the LASSO regression model takes double-standard error by constructing a penalty function. Concerning the characteristics of this method, we screened suitable and effective risk factors for T2DM patients with stroke in the LASSO regression analysis and selected 11 non-zero characteristic factors. We then obtained three models: foundation model, biochemical model, and integrated model, respectively, including basic physical indicators, biochemical indicators, and both indicators, and separately put into the multivariate logistic regression analysis. Variables selected through logistic regression analysis were considered of odds ratio (OR) and *P*-value with 95% confidence interval (CI), and the statistical significance levels were all two-sided. Based on the logistic regression results, we selected risk factors with the *P*-value of and <0.05 and constructed a nomogram prediction model. In this study, all the variables were selected. For the validation of the three models, we, respectively, calculated C index, receiver operating characteristic (ROC) curve, and dynamic component analysis (DCA) measurements based on the data from training set and validation set (16).

We used NRI and IDI to choose the best predictive model. NRI and IDI are two mutually complementary validation method to compare the accuracy and predictive ability of two prediction models, evaluating the effectiveness of index change compared with the old one. The difference between NRI and IDI is that the NRI only considers the improvement setting a certain cutoff point while the IDI inspects the overall improvement of the model. When NRI >0.1, the prediction model is improved, and if IDI >0.1, it indicates that this is an improvement and that the new model is better than the old model. The difference between NRI and IDI is that the NRI only considers the improvement when setting a certain cutoff point, while the IDI inspects the overall improvement of the model.

After selecting the best model, we applied the variables of the model to all the subjects in this study for internal validation to ensure the predictive ability of the model.

## Results

This study involved 4,335 T2DM patients, including 1,831 (42.24%) male participants and 2,504 (57.76%) female participants from seven communities in Shanghai. Among all the included T2DM patients, there were 379 patients (8.74%) with stroke and 3,956 patients (91.26%) without stroke. The average age of the participants was 64.54 ± 6.79 years. The prevalence of stroke among all participants was 8.74% (379 participants). The mean LDL-C and HDL-C levels in patients with stroke were 1.75 ± 0.48 and 1.47 ± 0.36 mmol/L and were 1.48 ± 0.46 and 1.73 ± 0.38 mmol/L separately in those without stroke. The median TG level was 2.00 (1.61, 2.59) mmol/L in patients with stroke and 1.24 (0.81, 1.91) mmol/L in those without stroke. The median HbA_1c_ and FBG levels of T2DM patients with stroke were separately 7.30% (6.60%, 8.30%) and 7.60 (6.35, 9.10) mmol/L, whereas those of patients without stroke were 6.57% (5.87%, 7.57%) and 7.03 (5.83, 8.63) mmol/L. In this study, the mean SBP and DBP levels were 148.38 ± 19.34 mmHg and 81.90 ± 10.76 mmHg in patients with stroke, whereas the median SBP and mean DBP levels were 132.00 (119.00, 145.00) mmHg and 77.18 ± 10.52 mmHg in patients without stroke. Among 4,335 T2DM patients, 2,880 (66.44%) people used antihypertensive drugs, 692 (15.96%) people used lipid-lowering drugs, 1,162 (26.81%) people used aspirin, and 772 (17.81%) people used insulin. Among the 3,956 T2DM patients without stroke, 2,702 (68.30%) patients used antihypertensive drugs, 562 (14.21%) patients used lipid-lowering drugs, 1,021 (25.81%) patients used aspirin, and 674 (17.04%) patients used insulin. Among the 379 stroke patients, 178 (46.97%) used antihypertensive drugs, 130 (34.30%) used lipid-lowering drugs, 141 (37.20%) used aspirin, and 98 (25.86%) used insulin.

For external verification, we divided two groups, training set (*n* = 3,252) and validation set (*n* = 1,083), at a ratio of 3:1. The training set composed of 1,380 (42.44%) male patients and 1,872 (57.56%) female patients, with average age of 64.46 ± 6.73 years. There were 284 patients (8.73%) complicated with stroke. In the validation set, 451 (41.64%) male patients and 632 (58.36%) female patients were included. The average age was 64.80 ± 6.95 years. Ninety-five patients (8.77%) were complicated with stroke. The detailed demographic and clinical characteristics are given in Table 1.

**Table 1:** Characteristics of the participants in different groups.

	**Total (*n* = 4,335)**	**Stroke (*n* = 379)**	**No stroke (*n* = 3,956)**	**Training set (*n* = 3,252)**	**Validation set (*n* = 1,083)**	***P***
Gender						0.648
Male	1,831 (42.24%)	176 (46.44%)	1,655 (41.84%)	1,380 (42.44%)	451 (41.64%)	
Female	2,504 (57.76%)	203 (53.56%)	2,301 (58.16%)	1,872 (57.56%)	632 (58.36%)	
Diagnosed stroke	379 (8.74%)			284 (8.73%)	95 (8.77%)	0.969
Age (years)	64.54 ± 6.79	66.97 ± 6.21	64.31 ± 6.79	64.46 ± 6.73	64.80 ± 6.95	0.146
Course (years)	9.00 (4.00, 14.00)	11.00 (6.00, 16.00)	8.00 (4.00, 14.00)	9.00 (4.00, 14.00)	9.00 (4.00, 14.00)	0.925
BMI (kg/m^2^)	24.23 ± 3.47	26.94 ± 3.36	23.97 ± 3.37	24.20 ± 3.44	24.32 ± 3.54	0.342
Hypertension						0.845
No	1,656 (38.20%)	85 (22.43%)	1,571 (39.71%)	1,245 (38.28%)	411 (37.95%)	
Yes	2,679 (61.80%)	294 (77.57%)	2,385 (60.29%)	2,007 (61.72%)	672 (62.05%)	
Hyperlipemia						0.912
No	2,716 (62.65%)	194 (51.19%)	2,522 (63.75%)	2,039 (62.70%)	677 (62.51%)	
Yes	1,619 (37.35%)	185 (48.81%)	1,434 (36.25%)	1,213 (37.30%)	406 (37.49%)	
Microvascular disease						0.191
No	2,155 (49.71%)	163 (43.01%)	1,992 (50.35%)	1,598 (49.14%)	557 (51.43%)	
Yes	2,180 (50.29%)	216 (56.99%)	1,964 (49.65%)	1,654 (50.86%)	526 (48.57%)	
Family history of DM						0.278
No	2,565 (59.17%)	225 (59.37%)	2,340 (59.15%)	1,909 (58.70%)	656 (60.57%)	
Yes	1,770 (40.83%)	154 (40.63%)	1,616 (40.85%)	1,343 (41.30%)	427 (39.43%)	
Smoking						0.184
No	3,544 (81.75%)	317 (83.64%)	3,227 (81.57%)	2,644 (81.30%)	900 (83.10%)	
Yes	791 (18.25%)	62 (16.36%)	729 (18.43%)	608 (18.70%)	183 (16.90%)	
Alcohol						0.094
No	3,288 (75.85%)	283 (74.67%)	3,005 (75.96%)	2,487 (76.48%)	801 (73.96%)	
Yes	1,047 (24.15%)	96 (25.33%)	951 (24.04%)	765 (23.52%)	282 (26.04%)	
SBP (mmHg)	133.00 (120.00, 147.00)	148.38 ± 19.34	132.00 (119.00, 145.00)	133 (120.00, 148.00)	133.00 (120.00, 146.00)	0.110
DBP (mmHg)	77.59 ± 10.62	81.90 ± 10.76	77.18 ± 10.52	77.73 ± 10.66	77.18 ± 10.51	0.139
FBG (mmol/L)	7.03 (5.73, 8.63)	7.60 (6.35, 9.10)	6.93 (5.73, 8.63)	7.03 (5.83, 8.63)	7.00 (5.70, 8.73)	0.871
PBG (mmol/L)	11.28 ± 4.81	12.45 ± 4.27	11.17 ± 4.84	7.70 (10.90, 14.40)	11.13 ± 4.92	0.246
HbA_1c_ (%)	6.57 (5.87, 7.67)	7.30 (6.60, 8.30)	6.57 (5.87, 7.57)	6.57 (5.87, 6.67)	6.57 (5.87, 7.67)	0.987
TC (mmol/L)	4.51 ± 1.09	4.86 ± 1.12	4.48 ± 1.08	4.50 ± 1.09	4.53 ± 1.06	0.463
TG (mmol/L)	1.32 (0.85, 2.00)	2.00 (1.61, 2.59)	1.24 (0.81, 1.91)	1.31 (0.85, 2.00)	1.34 (0.88, 1.99)	0.909
LDL-C (mmol/L)	1.51 ± 0.47	1.75 ± 0.48	1.48 ± 0.46	1.50 ± 0.47	1.52 ± 0.46	0.432
HDL-C (mmol/L)	1.71 ± 0.39	1.47 ± 0.36	1.73 ± 0.38	1.71 ± 0.39	1.72 ± 0.39	0.290
BUN (mmol/L)	5.81 (4.21, 6.25)	5.63 (4.72, 6.65)	5.14 (4.18, 6.20)	5.19 (4.24, 6.27)	5.14 (4.14, 6.18)	0.171
UA (μmol/L)	298.31 ± 79.89	343.43 ± 81.33	293.99 ± 78.39	298.66 ± 80.33	297.25 ± 78.52	0.614
eGFR (mL/min)	53.35 (33.05, 77.67)	72.37 (52.90, 97.59)	51.58 (31.49, 74.82)	53.31 (33.39, 77.74)	53.63 (32.44, 76.97)	0.794
ACR (mg/g)	23.33 (10.64, 59.08)	46.01 (30.95, 96.33)	20.36 (9.74, 54.60)	23.58 (10.64, 58.10)	22.71 (10.77, 60.75)	0.845

*Data are presented as n (%), mean ± SD, or median (IQR)*.

**Figure 1:**
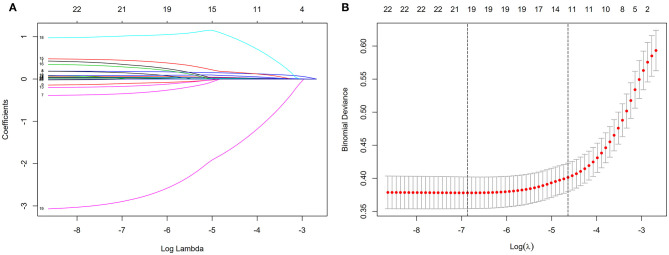
Demographic and clinical feature selection using the LASSO binary logistic regression model. **(A)** The selection of the best parameter (lambda) in the LASSO model uses 5-fold cross-validation with the lowest standard. The relationship curve between partial likelihood deviation (binomial deviation) and log(lambda) was plotted. Dotted vertical lines were drawn at the optimal values by using the minimum criteria and the 1 SE of the minimum criteria (the 1 – SE criteria). **(B)** LASSO coefficient profiles of the 11 features. A coefficient profile plot was produced against the log(lambda) sequence. Vertical line was drawn at the value selected using 5-fold cross-validation, where optimal lambda resulted in five features with non-zero coefficients. LASSO, least absolute shrinkage and selection operator; SE, standard error.

Through the analysis of literature search results and questionnaire results, 23 potential risk factors from physical examination indicators and biochemical examination indicators were included in the LASSO regression analysis (Figures 1A,B). We selected 11 non-zero characteristic variables in the LASSO regression results, including AGE, course, BMI, SBP, DBP, HbA_1c_, TG, LDL-C, HDL-C, UA, and eGFR (Table 2).

**Table 2:** Coefficients and lambda.min value of the LASSO regression.

**Factors**	**Coefficients**	**Lambda.min**
Age (years)	0.036	0.010
Course (years)	0.022	
BMI (kg/m^2^)	0.142	
SBP (mmHg)	0.027	
DBP (mmHg)	0.013	
HbA_1c_ (%)	0.170	
TG (mmol/L)	0.079	
LDL-C (mmol/L)	1.023	
HDL-C (mmol/L)	−1.689	
UA (μmol/L)	0.003	
eGFR (mL/min)	0.003	

**Figure 2:**
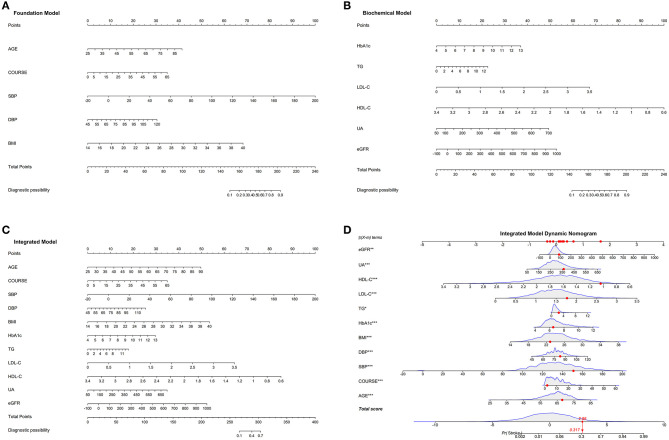
Developed nomograms of three T2DM combined stroke models. **(A)** Foundation model: the medication stroke nomogram for T2DM patients was developed in the cohort, with age, course, SBP, DBP, and BMI incorporated. **(B)** Biochemical model: the medication stroke nomogram for T2DM patients was developed in the cohort, with HbA_1c_, TG, LDL-C, HDL-C, UA, and eGFR incorporated. **(C)** Integrated model: the medication stroke nomogram for T2DM patients was developed in the cohort, with age, course, BMI, SBP, DBP, HbA_1c_, TG, LDL-C, HDL-C, UA, and eGFR incorporated. **(D)** An example of nomogram based on integrated model.

For external validation, three models were constructed. Foundation model, composed of basic information indicators and physical indicators, included AGE, course, SBP, DBP, and BMI (Figure 2A). Biochemical model consisted of biochemical indexes, including HbA_1c_, TG, LDL-C, HDL-C, UA, and eGFR (Figure 2B). Integrated model contained all the variables of the above two models (Figure 2C). To give a plain and clarified illustration of integrated model, an example of a T2DM patient demonstrated in Figure 2D. If the subject is at the age of 68 years, duration of 2 years, SBP of 151 mmHg, DBP of 83 mmHg, BMI of 22.84 kg/m^2^, HbA_1c_ of 6.5%, TG of 2.42 mmol/L, HDL-C of 1.04 mmol/L, LDL-C of 1.77 mmol/L, UA of 362 μmol/L, and eGFR of 85.89 mL/min, the probability of stroke is estimated to be 31.7%.

**Figure 3:**
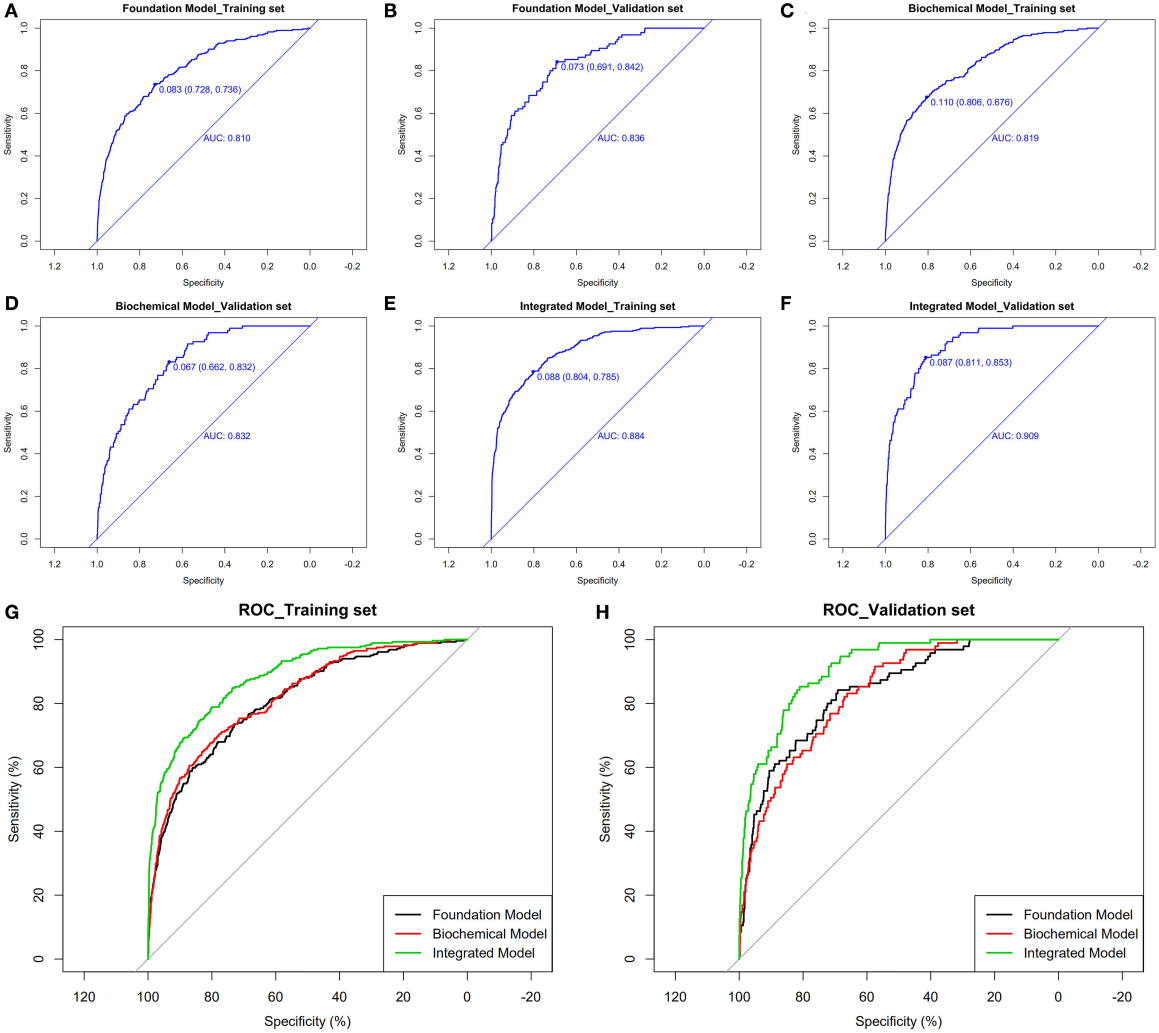
The pooled AUC of the ROC curve in training set and validation set. Foundation model **(A,B)**, biochemical model **(C,D)**, integrated model **(E,F)**: The *y* axis measures the net benefit. The dotted line represents the stroke incidence risk nomogram for T2DM patients. The thin solid line represents the assumption that all patients are diagnosed as stroke. The thick solid line represents the assumption that no patients are diagnosed as stroke. **(G,H)**: Integration of above decision curve analysis for the stroke incidence risk nomogram based on three models in training set and validation set.

**Figure 4:**
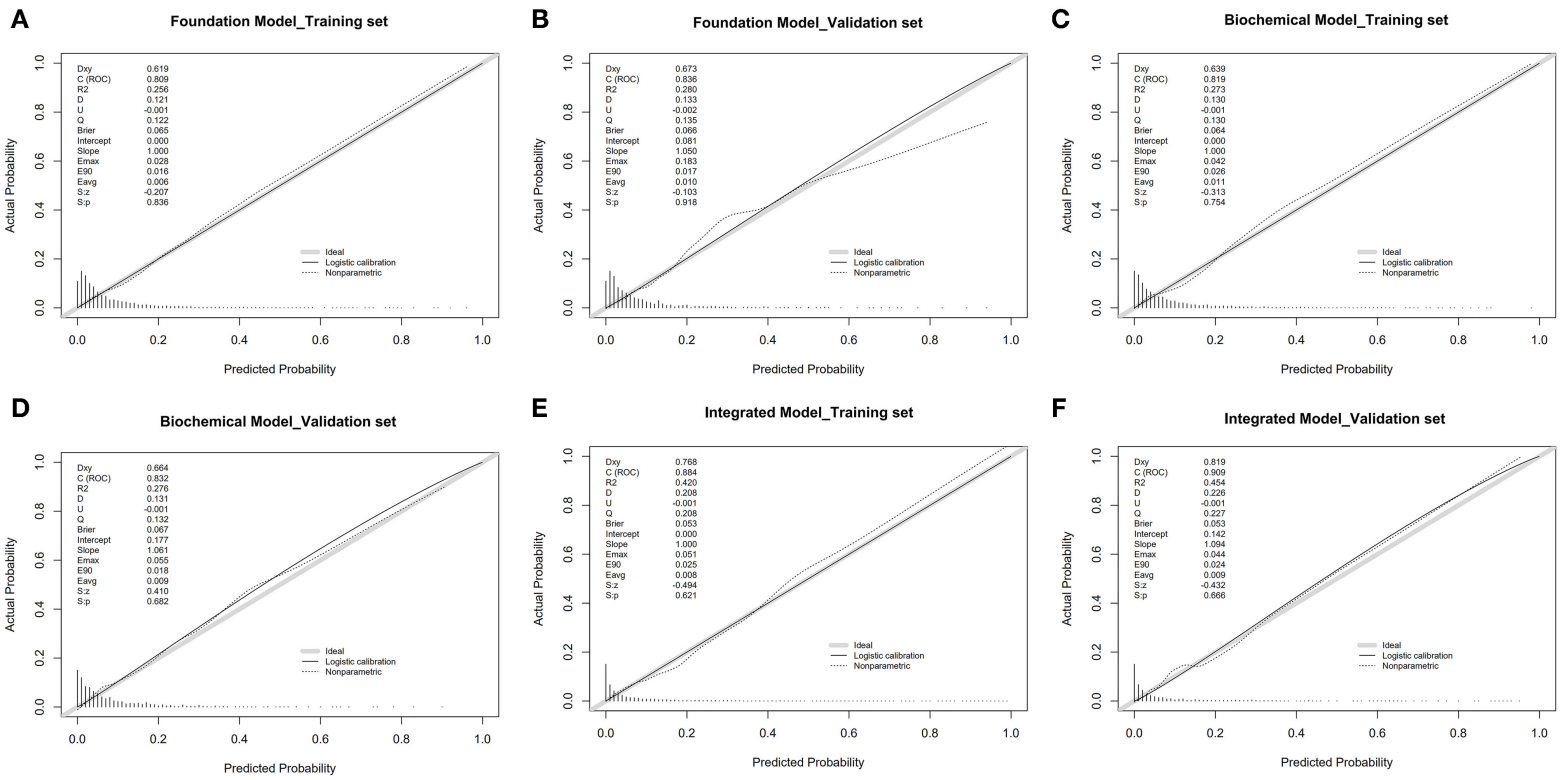
Calibration curves of the stroke incidence risk nomogram prediction in the array in training set and validation set. Foundation model **(A,B)**, biochemical model **(C,D)**, integrated model **(E,F)**: The *x* axis represents the predicted T2DM patients with stroke incidence risk. The *y* axis represents the actual diagnosed T2DM patients with stroke. The diagonal dotted line represents a perfect prediction by an ideal model. The solid line represents the performance of the nomogram, of which a closer fit to the diagonal dotted line represents a better prediction.

**Figure 5:**
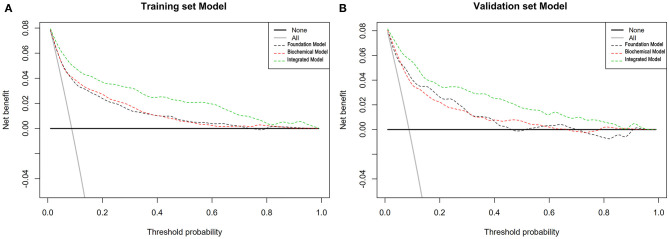
Decision curve analysis for the T2DM patients with stroke incidence risk nomogram based on three models in training set and validation set. **(A)** Training set and **(B)** validation set: The *y* axis means the true positive rate of the risk prediction of T2DM patients with stroke. The *x* axis means the false positive rate of the risk prediction of T2DM patients with stroke. The black line represents the performance of the nomogram of foundation model. The red line represents the performance of the nomogram of biochemical model. The green line represents the performance of the nomogram of integrated model.

Through logistic regression analysis, *p*-values of all risk characteristic factors were proved to be significant in the three models, respectively (Tables 3–5). The *C* index of foundation model, biochemical model, and integrated model were 0.810 (0.783–0.837), 0.819 (0.792–0.845), and 0.884 (0.863–0.905) (Table 6). Area under the curve (AUC) values of ROC for foundation model, biochemical model, and integrated model in training set (Figure 3G) were 0.810 (Figure 3A), 0.819 (Figure 3C), and 0.884 (Figure 3E) (Table 7), whereas in validation set (Figure 3H) correspondingly were 0.836 (Figure 3B), 0.832 (Figure 3D), and 0.909 (Figure 3F) (Table 7). Calibration plot indicated that S:P of foundation model, biochemical model, and integrated model in training set is 0.836 (Figure 4A), 0.754 (Figure 4C), and 0.621 (Figure 4E), whereas in validation set is, respectively, 0.918 (Figure 4B), 0.682 (Figure 4D), and 0.666 (Figure 4F). The DCA decision curve demonstrated that the threshold probability of foundation model, biochemical model, and integrated model in training set is 8–73%, 8–98% and ~8% (Figure 5A), whereas in validation set is 8–70, 8–90, and 8–95% (Figure 5B).

**Table 3:** Foundation model established by logistic regression analysis based on the training set.

	**β Coefficient**	**Wald test**	***P***	**OR (95% CI)**
Age (years)	0.052	4,696	<0.001	1.054 (1.031–1.078)
Course (years)	0.044	5.035	<0.001	1.045 (1.027–1.063)
SBP (mmHg)	0.037	10.798	<0.001	1.038 (1.031–1.045)
DBP (mmHg)	0.033	5.158	<0.001	1.034 (1.021–1.047)
BMI (kg/m^2^)	0.216	11.137	<0.001	1.241 (1.195–1.290)

**Table 4:** Biochemical Model established by logistic regression analysis based on the training set.

	**β Coefficient**	**Wald test**	***P***	**OR (95% CI)**
HbA_1c_ (%)	0.312	6.931	<0.001	1.366 (1.251–1.492)
TG (mmol/L)	0.132	2.817	0.005	1.141 (1.039–1.248)
LDL-C (mmol/L)	1.459	9.431	<0.001	4.301 (3.184–5.842)
HDL-C (mmol/L)	−2.711	−10.558	<0.001	0.066 (0.040–0.109)
UA (μmol/L)	0.006	6.556	<0.001	1.006 (1.004–1.008)
eGFR (mL/min)	0.004	2.835	0.005	1.004 (1.001–1.006)

**Table 5:** Integrated Model established by logistic regression analysis based on the training set.

	**β Coefficient**	**Wald test**	***P***	**OR (95% CI)**
Age (years)	0.063	5.144	<0.001	1.065 (1.040–1.091)
Course (years)	0.044	4.204	<0.001	1.045 (1.024–1.067)
SBP (mmHg)	0.037	9.380	<0.001	1.038 (1.030–1.046)
DBP (mmHg)	0.028	3.835	<0.001	1.028 (1.014–1.043)
BMI (kg/m^2^)	0.168	7.500	<0.001	1.183 (1.133–1.237)
HbA_1c_ (%)	0.270	5.038	<0.001	1.310 (1.179–1.455)
TG (mmol/L)	0.112	2.146	0.032	1.119 (1.007–1.237)
LDL-C (mmol/L)	1.508	8.766	<0.001	4.518 (3.24–6.354)
HDL-C (mmol/L)	−2.483	−9.024	<0.001	0.083 (0.048–0,142)
UA (μmol/L)	0.004	4.455	<0.001	1.004 (1.002–1.006)
eGFR (mL/min)	0.004	2.777	0.005	1.004 (1.001–1.007)

**Table 6:** *C* index in the array on the training set.

	***C* index** **(95% CI)**	**Dxy**	**aDxy**	**Variance**	***Z***	***P***	***n***
Foundation model	0.810 (0.783–0.837)	0.619	0.619	0.028	22.43	0	3,252
Biochemical model	0.819 (0.792–0.845)	0.639	0.639	0.027	23.88	0	3,252
Integrated model	0.884 (0.863–0.905)	0.768	0.768	0.021	36.38	0	3,252

**Table 7:** Comparison of ROC between different models using training set and validation set.

**Training set (*****n*** **=** **3,252)**						**Validation set (*****n*** **=** **1,083)**				
	**ROC A**	**ROC B**	**ROC C**	**A–C**	**B–C**	**ROC A**	**ROC B**	**ROC C**	**A–C**	**B–C**
AUC	80.950	81.927	88.380			83.642	83.196	90.942		
*Z*				−7.394	−6.684				−4.646	−5.048
***P***				<0.001	<0.001				<0.001	<0.001

**Figure 6:**
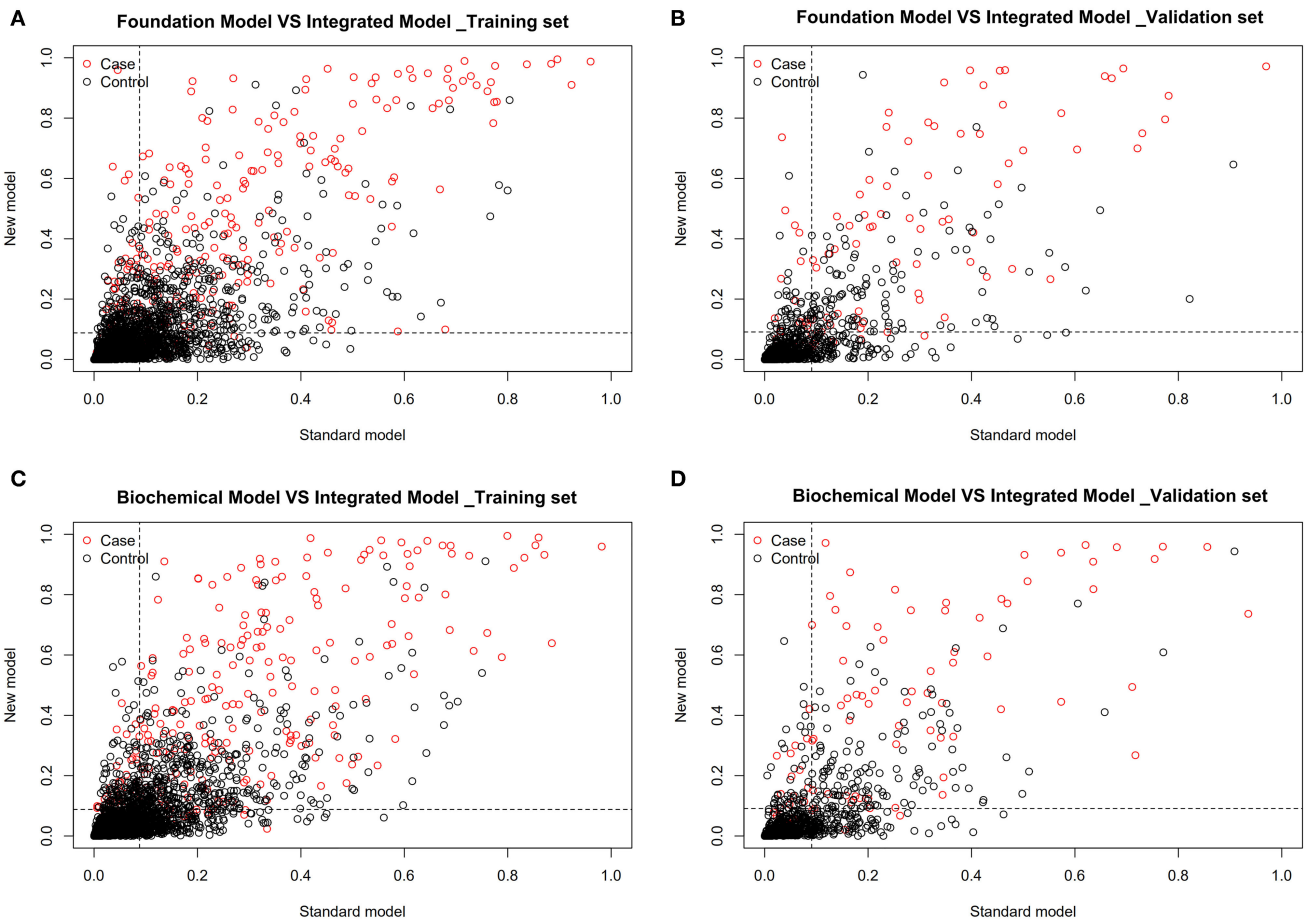
Model comparison based on NRI in training set [cutoff is 0.088 (0.804, 0.785)] and validation set [cutoff is 0.087 (0.811, 0.853)]. Foundation model vs. integrated model **(A,B)**: Integrated model is 0.131 better than foundation model in training set. Integrated model is 0.133 better than foundation model in validation set. Biochemical model vs. integrated model **(C,D)**: Integrated model is 0.113 better than biochemical model in training set. Integrated model is 0.118 better than biochemical model in validation set.

Through calculating the NRI, the cutoff in the training set was 0.088 (0.804, 0.785) (Figure 3E). Integrated model demonstrated to be 0.131 better than foundation model (Figure 6A) and 0.113 better than biochemical model (Figure 6C) (Table 8). In the validation set, the cutoff was 0.087 (0.811, 0.853) (Figure 3F). Integrated model was 0.133 better than foundation model (Figure 6B) and 0.118 better than biochemical model (Figure 6D) (Table 8). Through calculating the IDI in training set, integrated model was 0.148 (0.124, 0.172) better than foundation model and 0.139 (0.115, 0.164) better than biochemical model (Table 8). In the validation set, integrated model was 0.157 (0.115, 0.200) better than foundation model and 0.166 (0.120, 0.213) better than biochemical model (Table 8). Therefore, based on the above results, we can conclude that compared with foundation model and biochemical model; integrated model is improved, indicating that integrated model meets the clinical predictive modeling standards.

**Table 8:** Comparison of the prediction ability between different models through NRI and IDI.

	**Training set (*****n*** **=** **3,252)**		**Validation set (*****n*** **=** **1,083)**	
	**Foundation model **~** Integrated model**	**Biochemical model **~** Integrated model**	**Foundation model **~** Integrated model**	**Biochemical model **~** Integrated model**
NRI	0.131	0.113	0.133	0.118
*P*	0	0	0.004	<0.001
2.5% CI	0.078	0.065	0.080	0.068
97.5% CI	0.185	0.162	0.185	0.163
IDI	0.148	0.139	0.157	0.166
*P*	0	0	0	0
2.5% CI	0.124	0.115	0.115	0.120
97.5% CI	0.172	0.164	0.200	0.213

After obtaining integrated model, we verified on all the subjects included in this study with all the characteristic variables of integrated model and the variables proved to have a fairly good ability of predicting risk of stroke among T2DM patients. The result has been showed in Table 9.

**Table 9:** *C* index in the array in 4,335 T2DM patients.

***C*** **index** **(95% CI)**	**Dxy**	**aDxy**	**Variance**	***Z***	***P***	***n***
0.890 (0.873–0.907)	0.781	0.781	0.017	45.48	0	4,335

Based on the results, the integrated model was confirmed to have moderate predictive ability. To better aid prevention and treatment of T2DM patients with stroke clinically and in the community, we developed an online application that could predict quickly and directly. The URL of the application is https://doctorhu.shinyapps.io/T2DM_Stroke_DynNomapp/.

## Discussion

### Prevalence of Stroke, Differences in Clinical Characteristics, and Medication Conditions of T2DM Patients

The prevalence of stroke in T2DM patients was 8.74%, and those in training set and validation set were, respectively, 8.73 and 8.77% in the study, which were consistent with some other previous studies. In a national observational cohort study in Sweden, in 26,380 T2DM patients, 6.5% were diagnosed with a stroke with the stroke incidence rate of 10.12 events 1,000 person-years (17). A study including multivariate analysis conducted in Spain found 41.2% T2DM patients with atherothrombotic stroke and 35.1% with lacunar infarction (18). Shen et al. (19) performed a retrospective cohort study composed of 27,113 blacks and 40,431 whites with T2DM and found that 8,496 (12.57%) participants developed stroke during a mean follow-up period of 3 years. A Chinese study was conducted on 9,374 T2DM patients in total to establish a risk score system; among all the participants, 11.48% developed ischemic stroke with a mean follow-up of 8 years (12). Xuebing et al. (20) performed a study in Beijing, China, on 4,639 T2DM patients, and among all the subjects, the prevalence of stroke was 5.5%.

The biochemical indicator characteristics of the general population in this study, T2DM patients with stroke, were generally higher in the levels of clinical indicators, including LDL-C, TG, HbA_1c_, FBG, SBP, and DBP than those of patients without stroke, and HDL-C level was lower among patients with stroke, which were consistent with other studies. A study on Chinese T2DM patients indicated that LDL-C and TG were higher in patients with CVD, and HDL-C was lower than those without CVD (21). A study exploring risk factors of ischemic stroke on 2,769 DM patients found that the mean HbA_1c_ and FBG levels were significantly higher in patients with stroke when compared with patients without stroke (22). A Taiwanese study of 16,994 T2DM patients demonstrated that compared with those without stroke, patients with stroke were higher in the prevalence of hypertension with a rate of 74.5% (23).

During this study, more than two-thirds of patients took antihypertensive drugs, and nearly a third of patients use aspirin. A case-control study conducted in 32 countries/regions indicated that the occurrence of stroke is related to hypertension (24). A systematic review also showed that lowering blood pressure can significantly reduce various baseline blood pressure levels and vascular risk of complications (25). In our study, there were 2,702 T2DM patients without stroke taking antihypertensive drugs, accounting for 68.30% of all patients without stroke, which showed that taking antihypertensive drugs has significance on controlling blood pressure and then reducing the stroke incidence. According to a review comprehensively including randomized controlled trials of aspirin therapy, it is estimated that aspirin would reduce the risk of myocardial infarction and stroke by ~10% in DM patients, indicating that low-dose aspirin therapy (75–162 mg) would be reasonable for DM patients in the primary prevention for stroke (26).

### Risk Factors for T2DM Patients With Stroke

We utilized the nomogram in the study. A nomogram is a superior visual tool with the user-friendly display, precise calculation, and easy to understand and effective prognoses (27), which is expert in developing a graphic continuous scoring system based on incorporated related factors and calculating precisely the risk probability of adverse results according to individual characteristics (28). In terms of all the bright points, the nomogram was applied for predicting the risk of stroke incidence among T2DM patients and clinical evaluation and displayed decent predictive power through internal and external validation.

Eleven risk characteristic variables considered as factors affecting stroke incidence among T2DM patients in this study, including age, course, SBP, DBP, HDL-C, LDL-C, BMI, TG, eGFR, UA, HbA_1c_, were selected through LASSO and logistic regression analysis based on training set. Among three different models we established, integrated model incorporating all the 11 variables showed the best predictive ability through NRI and IDI validation, which displayed the necessity of each of the 11 risk factors in predicting the risk of stroke among T2DM patients. A risk study on T2DM patients with stroke obtained 14 risk factors, among which four risk factors, including age, disease course, blood pressure, and HbA_1c_ level, were consistent with this study (12).

According to the results, this study suggested that age and the course of diabetes in T2DM patients are important and immutable predictive risk factors for T2DM patients with stroke. Old age means the decline of the function of various tissues and organs of the body, pointing out that the risk of T2DM patients with stroke is affected by age (1). A study of 3,776 T2DM subjects identified age as an important risk factor (29). As the age of T2DM patients with stroke continues to increase, with the decline of physical function and the prolongation of the duration of diabetes, blood glucose fluctuations are obvious, exacerbating vascular endothelial damage and inflammatory stimuli, thereby accelerating the formation of stroke (30). Khalid Al-Rubeaan et al. (22) performed a study on ischemic stroke and its risk factors in a diabetic cohort in countries facing diabetes prevalence and showed the prevalence of ischemic stroke was 4.42% and was higher in the older age group with longer diabetes duration.

The result of this study illustrated that there was a positive correlation between BMI, TG, and stroke prevalence in T2DM patients. High BMI and TG indicate that patients are obese, having a higher possibility of blood lipid status. According to the American Heart Association, American Stroke Association, and many other global guidelines, maintaining a healthy weight is recommended as an important intervention for stroke outbreaks. The BMI, as an important measure of physical health, plays an important role in preventing the onset of disease in the brain of diabetic patients. For a cohort including patients with first-ever stroke, higher BMI was confirmed as an independent indicator for long-term survival according to a randomized controlled trial–based study on the effect of interventions targeting risk factors prevention (31). The study has shown that BMI has an impact on stroke risk in diabetic patients (11). A study of Chinese patients with T2DM showed that TG is a risk factor for stroke in T2DM patients and that female's elevated TG levels are more likely to be the risk factor to cause strokes than those of males (32). During the literature search, it was found that the results of some studies on BMI risk factors pointed out that the BMI of patients with type 2 diabetes was negatively related to the risk of stroke (33), which was consistent with the same results we obtained according to the available data.

The result of our study showed that there is a significantly positive association between the prevalence of stroke and blood pressure in patients with DM. The study has indicated that high blood pressure is the factor leading to increased stroke risk (34). A meta-analysis of randomized controlled trials comparing the effects including BP lowering on cardiovascular outcomes of DM patients concluded that BP-lowering treatment would significantly reduce cardiovascular risk in DM patients (35). According to the *Journal of the American Heart Association*, different from the cutoff point (BP ≥140/90 mmHg) for the diagnosis of hypertension in non-diabetic population, the diagnostic criteria of hypertension in diabetic patients are SBP ≥130 mmHg and/or DBP ≥80 mmHg (BP ≥130/80 mmHg) (36). Patients with T2DM often have comorbidities such as hypertension, obesity, and depression (37). Systolic blood pressure is one of the main diagnostic indicators of hypertension. Hypertension is the basis of arteriosclerosis, which can cause endothelial hyperplasia, sclerosis, vascular stenosis, and even occlusion. It is for this reason that strokes eventually occur. A study on high blood pressure showed that SBP and DBP are related to the occurrence of stroke (38). A review summarizing evidence mainly based on randomized controlled trials for the effect of BP management on the primary and secondary prevention of stroke determined that adequate BP lowering is of great significance and is expected to bring benefits for stroke prevention (39). Therefore, it is necessary to control SBP and DBP among T2DM patients.

HbA_1c_ is a parameter of sugar, indicating the 2- or 3-month mean level of blood glucose control and has a close link with the risk of diabetic complications (40). According to the result, glycemic control is essential as a preventable measure of stroke incidence for its influence on T2DM patients. A prospective cohort study conducted on 563 qualified T2DM patients showed that HbA_1c_ could affect the development of microvascular complications (41). A study in Pakistan that worked on the difference of HbA_1c_ values among diabetics and non-diabetics with stroke demonstrated that HbA_1c_ level was higher in the diabetic group (42). Through a Swedish study of 406,271 T2DM patients in total, T2DM patients were proved to have a higher risk of stroke and death with a lack of proper glycemic control, measured by the HbA_1c_ index (17). A systematic review including meta-analysis indicated that a rising HbA_1c_ level would be associated with the elevated risk of first-ever stroke, with average hazard ratios (95% CI) among DM cohorts of 1.17 (1.09, 1.25) as HbA_1c_ increased 1% (43). According to a study in Thailand based on T2DM patients with and without ischemic stroke, the risk of ischemic stroke would be raised 7.9–10.9 times with HbA_1c_ of 8–8.9% and higher (44).

Both LDL-C and HDL-C were considered as risk factors affecting stroke incidence of T2DM patients based on the result. A population-based retrospective cohort study on 144,271 Chinese T2DM patients found control of LDL-C was considerably related with 42% reduction of CVDs and should be given priority for treatment in primary care (45). Based on extensive clinical trials, a meta-analysis showed that the incidence rate of stroke among T2DM patients decreased by 21% with LDL-C level decreasing by 1 mmol/L (38.7 mg/dL) (46). High-density lipoprotein cholesterol is known for its antithrombotic influencing platelets, endothelial cells, and the blood coagulation–fibrinolysis system (47) and as a prevention factor of atherosclerosis. A meta-analysis on data of 61 studies indicated a strong association between HDL-C cholesterol and high risk of CVD and death (48). Through a retrospective cohort study and a mean follow-up of 3 years, including 67,544 T2DM patients, a significant adverse connection was found between HDL-C cholesterol among T2DM patients and the risk of total, ischemic, and hemorrhagic stroke (19). High-density lipoprotein cholesterol was an influencing factor involved in a Chinese retrospective cohort study aiming at establishing a predictive model of ischemic stroke among T2DM patients (12).

Uric acid is considered as a risk factor affecting the stroke incidence according to the result. Previous studies have shown that T2DM patients with stroke are usually considered to have a high level of serum UA. Through meta-analysis, a Chinese work proved that T2DM patients were vulnerable to cerebral infarction with a high level of serum UA, along with a finding that the UA level among T2DM patients with cerebral infarction was 29% higher than those without the symptom (49). A study on 1,017 non–insulin-independent DM patients with a 7-year follow-up for each patient demonstrated that a high UA level was considerably related to fatal and non-fatal stroke, thus proving the significant association between UA and stroke among T2DM patients (50). A study exploring links between serum UA level and cardiovascular complications in T2DM patients found that the hazard ratio (95% CI) of stroke was 1.19 (1.08, 1.31) with correspondence to every 59 μmol/L increase in UA level, indicating the serum UA level was related to the risk of stroke incidence among T2DM patients (51).

Estimated glomerular filtration rate is the indicator of renal function. In this study, eGFR was proved to be a risk factor of stroke in T2DM patients. Based on the discussions above, in a Roman study, eGFR was found to have a strong negative correlation to UA, thus indicating the association between eGFR and risk of stroke among DM patients in terms of the act of UA on stroke incidence (52). A study implemented in Poland found in a multivariate analysis that eGFR was considered as a risk factor of both diabetic and non-diabetic patients with ischemic stroke (53). A cross-sectional study conducted in Thailand based on 30,423 T2DM patients showed the association between decreased eGFR and increased risk of ischemic stroke, especially for those of eGFR <60 mL/min per 1.73 m^2^ (54).

### Limitations

However, our study still has a few limitations objectively. First, the number of subjects in our study is insufficient. In this study, all of the subjects were T2DM patients in seven communities in Shanghai, whereas still many patients were unable to participate in this study because of their serious condition. The prediction of risk factors for type 2 diabetes with stroke in other regions of China still requires more data to improve the prediction model. Second, there are relatively few indicators included in our study. Some indicators of lifestyle and socioeconomic factors should also be included in the study, such as smoking, drinking habits, education, income, and medication status (hypertensive drugs and lipid-lowering drugs). Also, we worked on the cross-sectional data without conducting subsequent related investigations. If the patient's indicators are followed up, the accuracy of this prediction model will be improved to a certain extent.

At the same time, current studies on the risk of stroke in T2DM patients in China mainly obtained data of hospitalized patients. There are insufficient epidemiological surveys conducted on T2DM patients in the community. At the beginning of this study, foundation model, biochemical model, and integrated model incorporating different risk factors were established at the step of external verification, which can be used to assess the risk of stroke in T2DM patients. Based on NRI and IDI, model C was finally identified as the best prediction model. That is to say, age, course, BMI, SBP, DBP, HbA_1c_, TG, HDL-C, LDL-C, UA, and eGFR are valuable predictors of risk. When applying the nomogram to T2DM patient evaluation, doctors must carry out health education from the perspective of medicine and skills guidance to help patients develop a healthier lifestyle.

## Conclusion

Based on a survey collecting basic information, physical data, and biochemical indicators of T2DM patients in seven communities in Shanghai, and processing-related data, this study established three predictive models of stroke risk for T2DM patients through risk factor analysis. To effectively apply the prediction model to T2DM patients and meet the needs of community management and clinical practice, tools including ROC, NRI, IDI, and internal verification were implemented in this study to determine the integrated model as the optimal and most accurate model among the three models.

## Data Availability Statement

Considering the privacy of patients, if readers have similar research and want to obtain data related to the article, they can contact the corresponding author, the corresponding research data can be obtained with permission.

## Ethics Statement

Shanghai Medical Ethics Society Committee waived the requirement for ethical approval for this study, which won support from Shanghai Municipal Health Commission before it began. The study was in accordance with the China Guideline for Type 2 Diabetes. All the subjects were carefully informed about the protocol and provided written informed consent before their inclusion in the study. This study protected the subject's anonymity. There is no identifiable information in this manuscript. Researchers kept all the questionnaires and signed informed consent forms.

## Author Contributions

RS was mainly responsible for data acquisition, including questionnaire design, recruitment and training of volunteers for questionnaire survey, communication with community health center affiliated to Shanghai University of traditional Chinese medicine. FH was responsible for the overall framework design of the paper, including experimental ideas, writing methods, data processing, building models, writing code by RStudio, completed community questionnaire recovery, and biochemical index test entry. TZ and HS were mainly responsible for the literature review, data interpretation, and manuscript compilation. All authors revised the manuscript and approved the current version submitted.

## Conflict of Interest

The authors declare that the research was conducted in the absence of any commercial or financial relationships that could be construed as a potential conflict of interest.

## Abbreviations

T2DM: Type 2 diabetes mellitus
DM: Diabetes mellitus
eGFR: Estimated glomerular filtration rate
PBG: Postprandial blood glucose
FBG: Fasting blood glucose
HbA_1c_: Glycosylated hemoglobin A_1c_
SBP: Systolic blood pressure
DBP: Diastolic blood pressure
LDL-C: Low-density lipoprotein cholesterol
HDL-C: High-density lipoprotein cholesterol
TC: Total cholesterol
TG: Triglyceride
BMI: Body mass index
UA: Uric acid
BUN: Blood urea nitrogen
ACR: Ratio of urinary microalbumin to uric creatinine
NRI: Net reclassification improvement
IDI: Integrated discrimination improvement
LASSO: Least absolute shrinkage and selection operator
OR: Odds ratio
CI: Confidence interval
ROC: Receiver operating characteristic
DCA: Decision curve analysis
SE: Standard error.

## References

[1] Akin S Boluk C Prevalence of comorbidities in patients with type-2 diabetes mellitus. Prim Care Diabetes. (in press) 10.1016/j.pcd.2019.12.006 31902582

[2] Dal Canto E Ceriello A Ryden L Ferrini M Hansen TB Schnell O. Diabetes as a cardiovascular risk factor: An overview of global trends of macro and micro vascular complications. Euro J Prev Cardiol. (2019) 26:25–32. 10.1177/2047487319878371 31722562

[3] Altemimi MT Hashim AR. Acute stroke in diabetes mellitus: a prospective observational study evaluating the course and short-term outcome in Basrah, Southern Iraq. Cureus. (2019) 11:e6017. 10.7759/cureus.6017 31824785 PMC6886642

[4] Gujjar AR. Diabetes and stroke: more than just accelerated atherosclerosis? Sultan Qaboos Univ Med J. (2018) 18:e261–e3. 10.18295/squmj.2018.18.03.001 30607264 PMC6307639

[5] Almdal T Scharling H Jensen JS Vestergaard H. The independent effect of type 2 diabetes mellitus on ischemic heart disease, stroke, and death - A population-based study of 13000 men and women with 20 years of follow-up. Arch Intern Med. (2004) 164:1422–6. 10.1001/archinte.164.13.1422 15249351

[6] Jorgensen HS Nakayama H Raaschou HO Olsen TS. Stroke in patients with diabetes - the copenhagen-stroke-study. Stroke. (1994) 25:1977–84. 10.1161/01.STR.25.10.1977 8091441

[7] Burchfiel CM Curb JD Rodriguez BL Abbott RD Chiu D Yano K. Glucose intolerance and 22-year stroke incidence. the honolulu heart program. Stroke. (1994) 25:951–7. 10.1161/01.STR.25.5.951 8165689

[8] Janghorbani M Hu FB Willett WC Li TY Manson JE Logroscino G. Prospective study of type 1 and type 2 diabetes and risk of stroke subtypes: the Nurses' Health study. Diabetes Care. (2007) 30:1730–5. 10.2337/dc06-2363 17389335

[9] Zhang XD Chen YR Ge L Ge ZM Zhang YH. Features of stroke in Chinese diabetes patients: a hospital-based study. J Int Med Res. (2007) 35:540–6. 10.1177/147323000703500414 17697532

[10] Bhattacharyya OK Shah BR Booth GL. Management of cardiovascular disease in patients with diabetes: the 2008. Canadian Diabetes Association guidelines. Canad Med Assoc J. (2008) 179:920–6. 10.1503/cmaj.080554 18801878 PMC2565732

[11] Wang S Chen J Wang Y Yang Y Zhang D Liu C. Cigarette smoking is negatively associated with the prevalence of Type 2 diabetes in middle-aged men with normal weight but positively associated with stroke in men. J Diab Res. (2019) 2019:1853018. 10.1155/2019/1853018 31612146 PMC6755302

[12] Li T-C Wang H-C Li C-I Liu C-S Lin W-Y Lin C-H. Establishment and validation of a prediction model for ischemic stroke risks in patients with type 2 diabetes. Diabetes Res Clin Pract. (2018) 138:220–8. 10.1016/j.diabres.2018.01.034 29458073

[13] 13 Bos MJ Koudstaal PJ Hofman A Witteman JCM Breteler MMB. Uric acid is a risk factor for myocardial infarction and stroke: the Rotterdam study. Stroke. (2006) 37:1503–7. 10.1161/01.STR.0000221716.55088.d4 16675740

[14] Gavin JR Alberti K Davidson MB DeFronzo RA Drash A Gabbe SG Report of the expert committee on the diagnosis and classification of diabetes mellitus. Diabetes Care. (1997) 20:1183–97. 10.2337/diacare.20.7.1183 9203460

[15] Li W Xie B Qiu S Huang X Chen J Wang X. Non-lab and semi-lab algorithms for screening undiagnosed diabetes: a cross-sectional study. Ebiomedicine. (2018) 35:307–16. 10.1016/j.ebiom.2018.08.009 30115607 PMC6154869

[16] Lyu J Li Z Wei H Liu D Chi X Gong D-W. A potent risk model for predicting new-onset acute coronary syndrome in patients with type 2 diabetes mellitus in Northwest China. Acta Diabetol. (2020) 57:705–13. 10.1007/s00592-020-01484-x 32008161 PMC7220880

[17] Zabala A Darsalia V Holzmann MJ Franzen S Svensson A-M Eliasson B. Risk of first stroke in people with type 2 diabetes and its relation to glycaemic control: a nationwide observational study. Diabetes Obes Metabol. (2020) 22:182–90. 10.1111/dom.13885 31576643

[18] Arboix A Rivas A Garcia-Eroles L de Marcos L Massons J Oliveres M. Cerebral infarction in diabetes: clinical pattern, stroke subtypes, and predictors of in-hospital mortality. BMC Neurol. (2005) 5:9. 10.1186/1471-2377-5-9 15833108 PMC1097737

[19] Shen Y Shi L Nauman E Katzmarzyk PT Price-Haywood EG Bazzano AN. Inverse association between HDL (high-density lipoprotein) cholesterol and stroke risk among patients with Type 2 Diabetes Mellitus. Stroke. (2019) 50:291–7. 10.1161/STROKEAHA.118.023682 30626289 PMC6349480

[20] Zhang XB Mu YM Yan WH Ba JM Li HM. Prevalence of stroke and metabolic disorders in the middle-aged and elderly Chinese with type 2 diabetes. Chin Med J. (2014) 127:3537–42. 25316225

[21] Ren Y Jin N Hong T Mu Y Guo L Ji Q. Interactive effect of serum uric acid and total bilirubin for cardiovascular disease in Chinese patients with type 2 diabetes. Sci Rep. (2016) 6:36437. 10.1038/srep36437 27805038 PMC5090353

[22] Al-Rubeaan K Al-Hussain F Youssef AM Subhani SN Al-Sharqawi AH Ibrahim HM. Ischemic stroke and its risk factors in a registry-based large cross-sectional diabetic cohort in a country facing a diabetes epidemic. J Diabetes Res. (2016) 2016:4132589. 10.1155/2016/4132589 26989695 PMC4771899

[23] Tseng CH Chong CK Sheu JJ Wu TH Med CP. Prevalence and risk factors for stroke in Type 2 diabetic patients in Taiwan: a cross-sectional survey of a national sample by telephone interview. Diabetic Med. (2005) 22:477–82. 10.1111/j.1464-5491.2005.01452.x 15787676

[24] O'Donnell MJ Chin SL Rangarajan S Xavier D Liu L Zhang H. Global and regional effects of potentially modifiable risk factors associated with acute stroke in 32 countries (INTERSTROKE): a case-control study. Lancet. (2016) 388:761–75. 10.1016/S0140-6736(16)30506-2 27431356

[25] Ettehad D Emdin CA Kiran A Anderson SG Callender T Rahimi K. Blood pressure lowering for prevention of cardiovascular disease and death: a systematic review and meta-analysis. Euro Heart J. (2016) 37:639. 10.1016/S0140-6736(15)01225-8 26724178

[26] Pignone M Williams CD. Aspirin for primary prevention of cardiovascular disease in diabetes mellitus. Nat Rev Endocrinol. (2010) 6:619–28. 10.1038/nrendo.2010.169 20856266 PMC3145323

[27] Wei L Champman S Li X Li X Li S Chen R. Beliefs about medicines and non-adherence in patients with stroke, diabetes mellitus and rheumatoid arthritis: a cross-sectional study in China. BMJ Open. (2017) 7:e017293. 10.1136/bmjopen-2017-017293 28982826 PMC5640055

[28] Sun C Li X Song B Chen X Nyame L Liu Y. A NADE nomogram to predict the probability of 6-month unfavorable outcome in Chinese patients with ischemic stroke. BMC Neurol. (2019) 19:274. 10.1186/s12883-019-1464-6 31699038 PMC6839074

[29] Davis TME Millns H Stratton IM Holman RR Turner RC Grp UKPDS. Risk factors for stroke in type 2 diabetes mellitus - United Kingdom prospective diabetes study (UKPDS) 29. Arch Intern Med. (1999) 159:1097–103. 10.1001/archinte.159.10.1097 10335687

[30] Banerjee C Moon YP Paik MC Rundek T Mora-McLaughlin C Vieira JR. Duration of diabetes and risk of ischemic stroke: the Northern Manhattan study. Stroke. (2012) 43:1212–7. 10.1161/STROKEAHA.111.641381 22382158 PMC3336044

[31] Hagberg G Fure B Sandset EC Thommessen B Ihle-Hansen H Oksengard AR. Long-term effects on survival after a 1-year multifactorial vascular risk factor intervention after stroke or TIA: secondary analysis of a randomized controlled trial, a 7-year follow-up study. Vasc Health Risk Manag. (2019) 15:11–18. 10.2147/VHRM.S191873 30799926 PMC6369929

[32] Cui R Qi Z Zhou L Li Z Li Q Zhang J. Evaluation of serum lipid profile, body mass index, and waistline in Chinese patients with type 2 diabetes mellitus. Clin Interv Aging. (2016) 11:445–52. 10.2147/CIA.S104803 27143868 PMC4841420

[33] Li W Katzmarzyk PT Horswell R Zhang Y Zhao W Wang Y. Body mass index and stroke risk among patients with type 2 diabetes mellitus. Stroke. (2015) 46:164. 10.1161/STROKEAHA.114.006718 25468880 PMC4276457

[34] Hu G Sarti C Jousilahti P Peltonen M Qiao Q Antikainen R. The impact of history of hypertension and type 2 diabetes at baseline on the incidence of stroke and stroke mortality. Stroke. (2005) 36:2538–43. 10.1161/01.STR.0000190894.30964.75 16282538

[35] Thomopoulos C Parati G Zanchetti A. Effects of blood-pressure-lowering treatment on outcome incidence in hypertension: 10 – should blood pressure management differ in hypertensive patients with and without diabetes mellitus? Overview and meta-analyses of randomized trials. J Hypertens. (2017) 35:922–44. 10.1097/HJH.0000000000001276 28141660

[36] White WB Jalil F Cushman WC Bakris GL Bergenstal R Heller SR. Average clinician-measured blood pressures and cardiovascular outcomes in patients with Type 2 diabetes mellitus and ischemic heart disease in the EXAMINE trial. J Am Heart Assoc. (2018) 7:e009114. 10.1161/JAHA.118.009114 30371278 PMC6474950

[37] Campbell RK. Type 2 diabetes: Where we are today: An overview of disease burden, current treatments, treatment strategies. J Am Pharm Assoc. (2009) 49:S3–9. 10.1331/JAPhA.2009.09077 19801365

[38] Willmot M Leonardi-Bee J Bath PMW. High blood pressure in acute stroke and subsequent outcome - a systematic review. Hypertension. (2004) 43:18–24. 10.1161/01.HYP.0000105052.65787.35 14662649

[39] Hong K-S. Blood pressure management for stroke prevention and in acute stroke. J Stroke. (2017) 19:152–65. 10.5853/jos.2017.00164 28592775 PMC5466289

[40] Nalysnyk L Hernandez-Medina M Krishnarajah G. Glycaemic variability and complications in patients with diabetes mellitus: evidence from a systematic review of the literature. Diabetes Obes Metab. (2010) 12:288–98. 10.1111/j.1463-1326.2009.01160.x 20380649

[41] Yozgatli K Lefrandt JD Noordzij MJ Oomen PHN Brouwer T Jager J. Accumulation of advanced glycation end products is associated with macrovascular events and glycaemic control with microvascular complications in Type 2 diabetes mellitus. Diab Med. (2018) 35:1242–8. 10.1111/dme.13651 29687658

[42] Nomani AZ Nabi S Ahmed S Iqbal M Rajput HM Rao S. High HbA1c is associated with higher risk of ischaemic stroke in Pakistani population without diabetes. Stroke Vasc Neurol. (2016) 1:133–9. 10.1136/svn-2016-000018 28959475 PMC5435196

[43] Mitsios JP Ekinci EI Mitsios GP Churilov L Thijs V. Relationship between glycated hemoglobin and stroke risk: a systematic review and meta-analysis. J Am Heart Assoc. (2018) 7:53. 10.1161/JAHA.117.007858 29773578 PMC6015363

[44] Chaveepojnkamjorn W Boonrasri W Viwatwongkasem C Siri S Kriengkaisakda W Relationship between hemoglobin A1c and ischemic stroke among patients with type-2 diabetes. Southeast Asian J Trop Med Public Health. (2019) 50:935–41. 10.1007/s13410-020-00806-7

[45] Wan EYF Fung CSC Yu EYT Chin WY Fong DYT Chan AKC. Effect of multifactorial treatment targets and relative importance of hemoglobin A1c, blood pressure, and low-density lipoproteincholesterol on cardiovascular diseases in chinese primary care patients with Type 2 diabetes mellitus: a population-based retrospective cohort study. J Am Heart Assoc. (2017) 6:13. 10.1161/JAHA.117.006400 28862945 PMC5586469

[46] 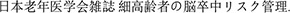 (2017) 54:524–30. 10.3143/geriatrics.54.524

[47] Annema W von Eckardstein A Kovanen PT. HDL and atherothrombotic vascular disease. Handb Exp Pharmacol. (2015) 224:369–403. 10.1007/978-3-319-09665-0_11 25522995

[48] Lewington S Whitlock G Clarke R Sherliker P Emberson J Halsey J. Blood cholesterol and vascular mortality by age, sex, and blood pressure: a meta-analysis of individual data from 61 prospective studies with 55000 vascular deaths. Lancet. (2008). 372:292. 10.1016/S0140-6736(07)61778-4 18061058

[49] Du L Ma JH Zhang XN. Higher serum uric acid may contribute to cerebral infarction in patients with type 2 diabetes mellitus: a meta-analysis. J Mol Neurosci. (2017) 61:25–31. 10.1007/s12031-016-0848-y 27696108

[50] Lehto S Niskanen L Ronnemaa T Laakso M. Serum uric acid is a strong predictor of stroke in patients with non–insulin-dependent diabetes mellitus. Stroke. (1998) 29:635–9. 10.1161/01.STR.29.3.635 9506605

[51] Shao YX Shao H Sawhney MS Shi LZ. Serum uric acid as a risk factor of all-cause mortality and cardiovascular events among type 2 diabetes population: Meta-analysis of correlational evidence. J Diab Complic. (2019) 33:7. 10.1016/j.jdiacomp.2019.07.006 31439471

[52] Gaita L Timar R Lupascu N Roman D Albai A Potre O. The impact of hyperuricemia on cardiometabolic risk factors in patients with diabetes mellitus: a cross-sectional study. Diabetes Metab Syndr Obesity-Targets Ther. (2019) 12:2003–10. 10.2147/DMSO.S222570 31632111 PMC6781154

[53] Snarska KK Bachorzewska-Gajewska H Kapica-Topczewska K Drozdowski W Chorazy M Kulakowska A. Hyperglycemia and diabetes have different impacts on outcome of ischemic and hemorrhagic stroke. Arch Med Sci. (2017) 13:100–8. 10.5114/aoms.2016.61009 28144261 PMC5206364

[54] Kaewput W Thongprayoon C Rangsin R Mao MA Satirapoj B Cheungpasitporn W. The association between renal function and neurological diseases in type 2 diabetes: a multicenter nationwide cross-sectional study. Hosp Pract. (1995) 47:46–52. 10.1080/21548331.2019.1549916 30445880

